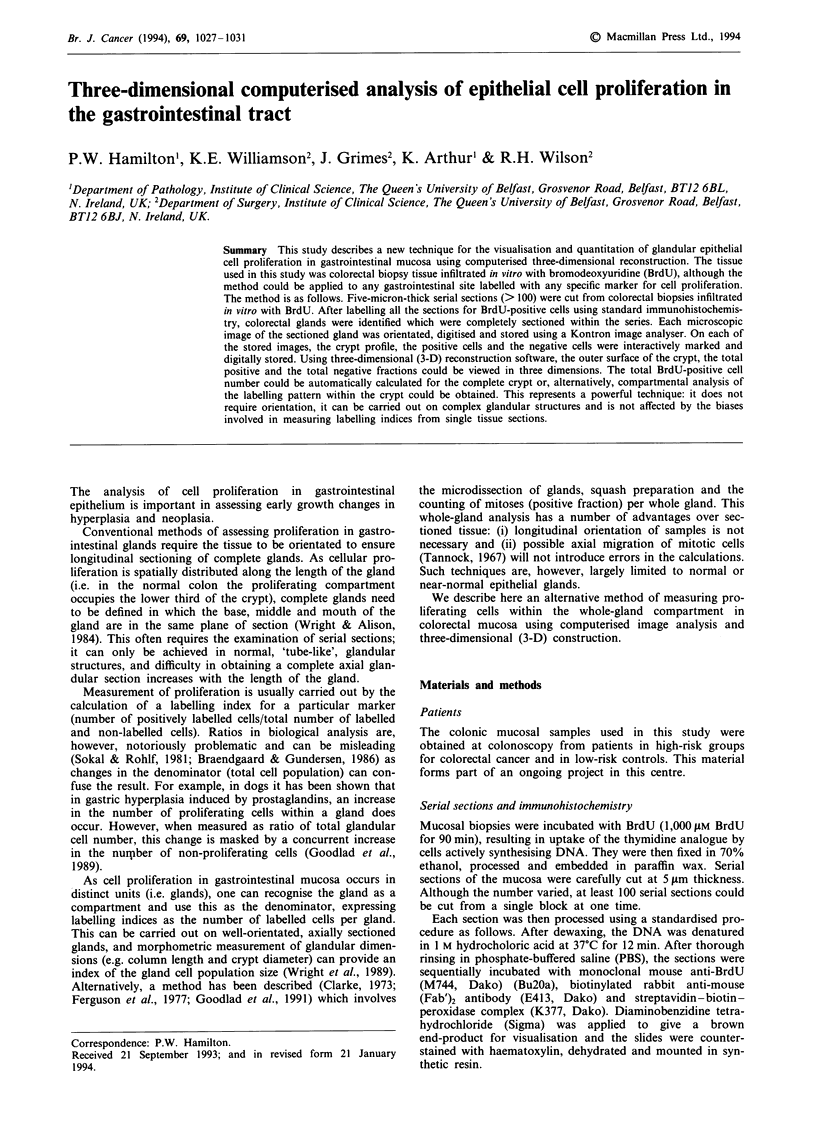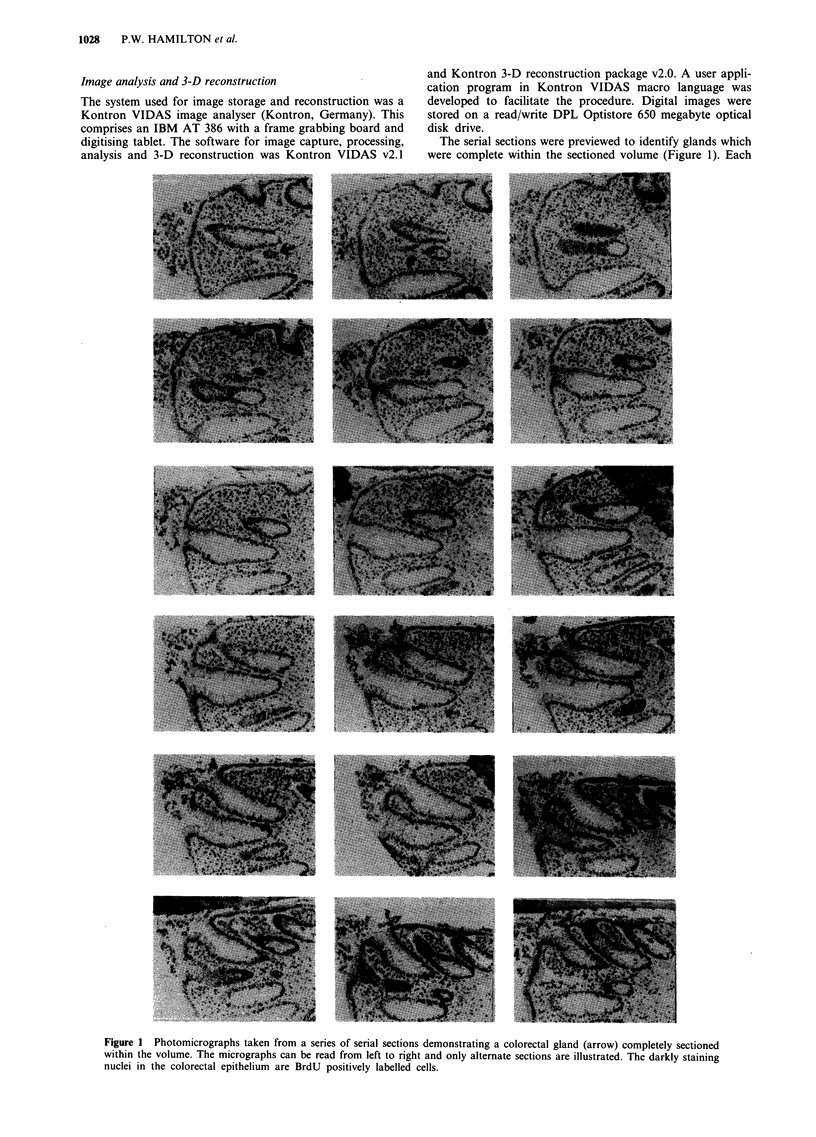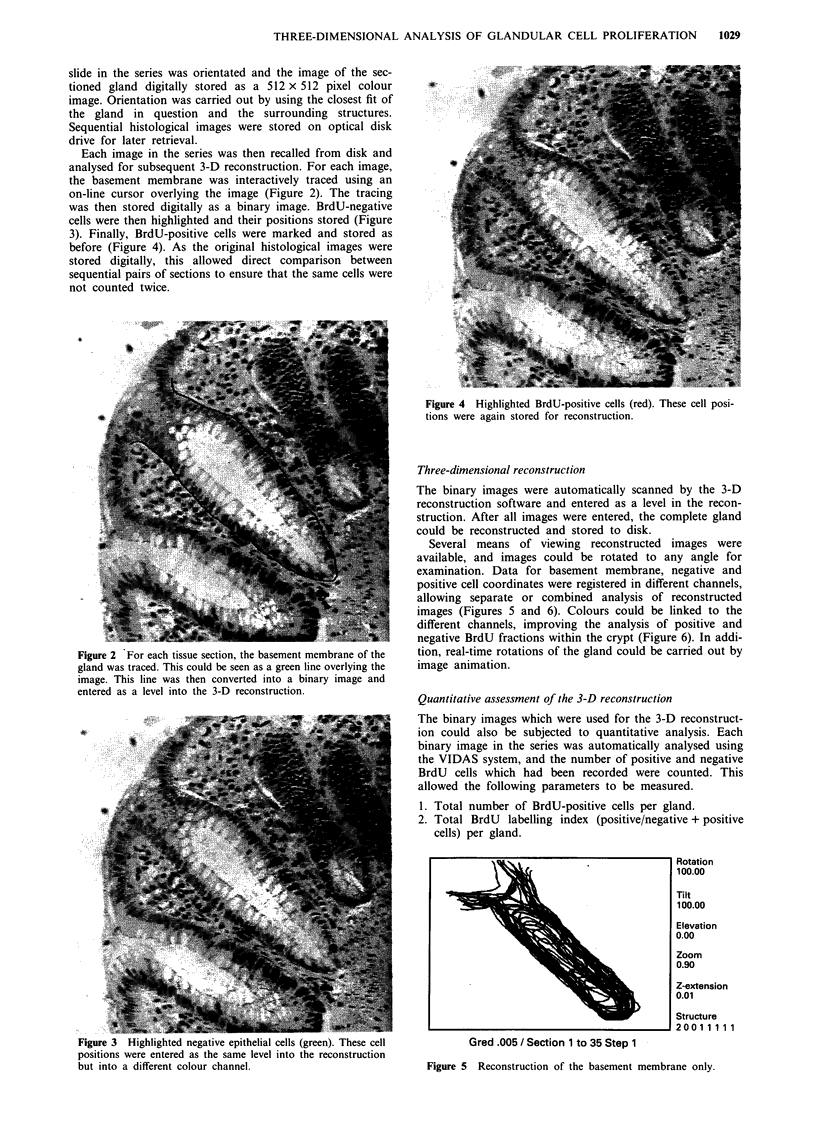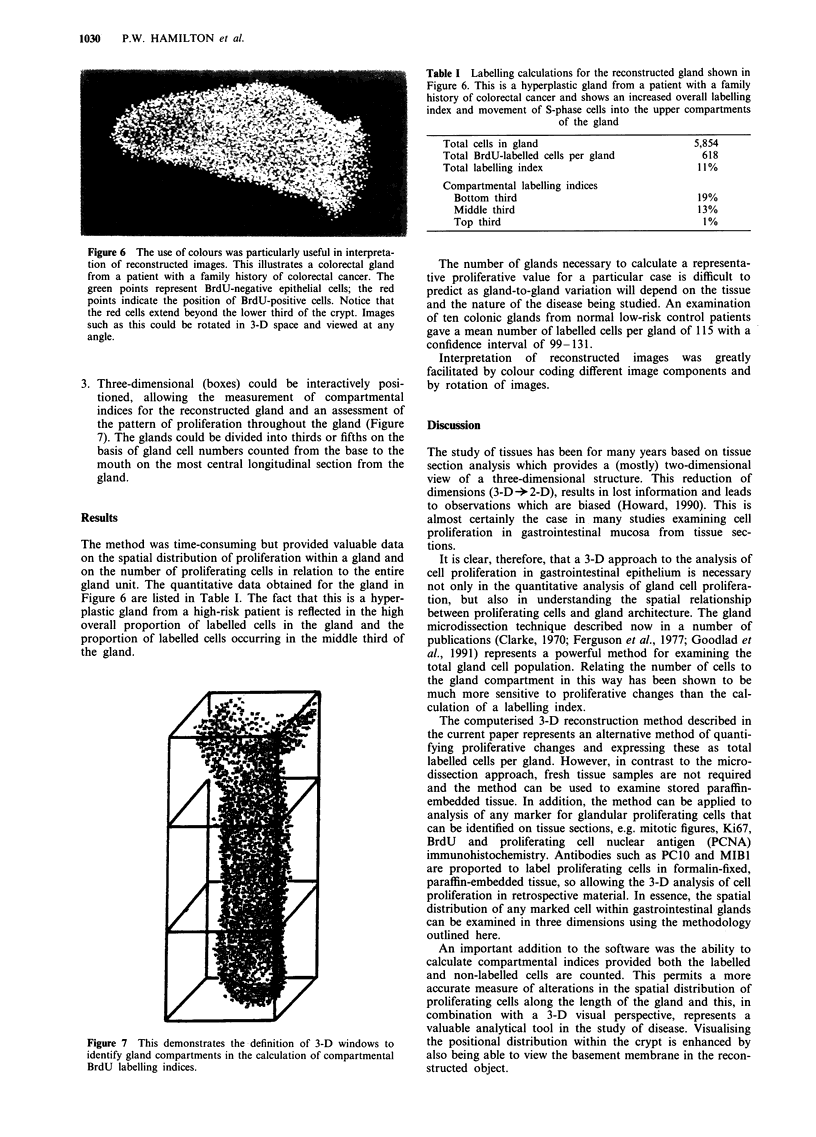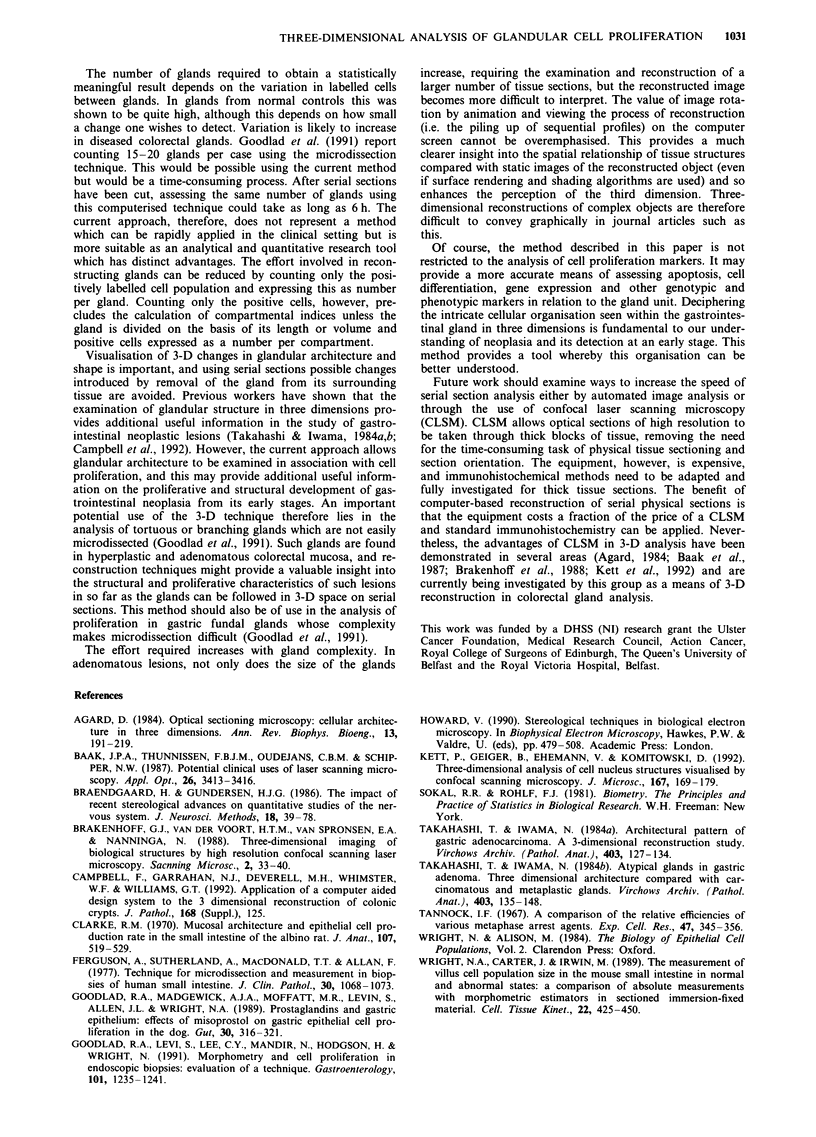# Three-dimensional computerised analysis of epithelial cell proliferation in the gastrointestinal tract.

**DOI:** 10.1038/bjc.1994.202

**Published:** 1994-06

**Authors:** P. W. Hamilton, K. E. Williamson, J. Grimes, K. Arthur, R. H. Wilson

**Affiliations:** Department of Pathology, Queen's University of Belfast, N. Ireland, UK.

## Abstract

**Images:**


Br. J. Cancer (1994), 69, 1027-1031 © Macmillan Press Ltd., 1994

### Three-dimensional computerised analysis of epithelial cell proliferation in the gastrointestinal tract

P.W. Hamilton[1], K.E. Williamson[2], J. Grimes[2], K. Arthur[1] & R.H. Wilson[2]

[1]*Department of Pathology, Institute of Clinical Science, The Queen's University of Belfast, Grosvenor Road, Belfast, BT12 6BL, N. Ireland, UK;* [2]*Department of Surgery, Institute of Clinical Science, The Queen's University of Belfast, Grosvenor Road, Belfast, BT12 6BJ, N. Ireland, UK.*

**Summary** This study describes a new technique for the visualisation and quantitation of glandular epithelial cell proliferation in gastrointestinal mucosa using computerised three-dimensional reconstruction. The tissue used in this study was colorectal biopsy tissue infiltrated *in vitro* with bromodeoxyuridine (BrdU), although the method could be applied to any gastrointestinal site labelled with any specific marker for cell proliferation. The method is as follows. Five-micron-thick serial sections (> 100) were cut from colorectal biopsies infiltrated *in vitro* with BrdU. After labelling all the sections for BrdU-positive cells using standard immunohistochemistry, colorectal glands were identified which were completely sectioned within the series. Each microscopic image of the sectioned gland was orientated, digitised and stored using a Kontron image analyser. On each of the stored images, the crypt profile, the positive cells and the negative cells were interactively marked and digitally stored. Using three-dimensional (3-D) reconstruction software, the outer surface of the crypt, the total positive and the total negative fractions could be viewed in three dimensions. The total BrdU-positive cell number could be automatically calculated for the complete crypt or, alternatively, compartmental analysis of the labelling pattern within the crypt could be obtained. This represents a powerful technique: it does not require orientation, it can be carried out on complex glandular structures and is not affected by the biases involved in measuring labelling indices from single tissue sections.

The analysis of cell proliferation in gastrointestinal epithelium is important in assessing early growth changes in hyperplasia and neoplasia.

Conventional methods of assessing proliferation in gastrointestinal glands require the tissue to be orientated to ensure longitudinal sectioning of complete glands. As cellular proliferation is spatially distributed along the length of the gland (i.e. in the normal colon the proliferating compartment occupies the lower third of the crypt), complete glands need to be defined in which the base, middle and mouth of the gland are in the same plane of section (Wright & Alison, 1984). This often requires the examination of serial sections; it can only be achieved in normal, 'tube-like', glandular structures, and difficulty in obtaining a complete axial glandular section increases with the length of the gland.

Measurement of proliferation is usually carried out by the calculation of a labelling index for a particular marker (number of positively labelled cells/total number of labelled and non-labelled cells). Ratios in biological analysis are, however, notoriously problematic and can be misleading (Sokal & Rohlf, 1981; Braendgaard & Gundersen, 1986) as changes in the denominator (total cell population) can confuse the result. For example, in dogs it has been shown that in gastric hyperplasia induced by prostaglandins, an increase in the number of proliferating cells within a gland does occur. However, when measured as ratio of total glandular cell number, this change is masked by a concurrent increase in the number of non-proliferating cells (Goodlad *et al.*, 1989).

As cell proliferation in gastrointestinal mucosa occurs in distinct units (i.e. glands), one can recognise the gland as a compartment and use this as the denominator, expressing labelling indices as the number of labelled cells per gland. This can be carried out on well-orientated, axially sectioned glands, and morphometric measurement of glandular dimensions (e.g. column length and crypt diameter) can provide an index of the gland cell population size (Wright *et al.*, 1989). Alternatively, a method has been described (Clarke, 1973; Ferguson *et al.*, 1977; Goodlad *et al.*, 1991) which involves the microdissection of glands, squash preparation and the counting of mitoses (positive fraction) per whole gland. This whole-gland analysis has a number of advantages over sectioned tissue: (i) longitudinal orientation of samples is not necessary and (ii) possible axial migration of mitotic cells (Tannock, 1967) will not introduce errors in the calculations. Such techniques are, however, largely limited to normal or near-normal epithelial glands.

We describe here an alternative method of measuring proliferating cells within the whole-gland compartment in colorectal mucosa using computerised image analysis and three-dimensional (3-D) construction.

### Materials and methods

### *Patients*

The colonic mucosal samples used in this study were obtained at colonoscopy from patients in high-risk groups for colorectal cancer and in low-risk controls. This material forms part of an ongoing project in this centre.

### *Serial sections and immunohistochemistry*

Mucosal biopsies were incubated with BrdU (1,000 μM BrdU for 90 min), resulting in uptake of the thymidine analogue by cells actively synthesising DNA. They were then fixed in 70% ethanol, processed and embedded in paraffin wax. Serial sections of the mucosa were carefully cut at 5 μm thickness. Although the number varied, at least 100 serial sections could be cut from a single block at one time.

Each section was then processed using a standardised procedure as follows. After dewaxing, the DNA was denatured in 1 M hydrocholoric acid at 37°C for 12 min. After thorough rinsing in phosphate-buffered saline (PBS), the sections were sequentially incubated with monoclonal mouse anti-BrdU (M744, Dako) (Bu20a), biotinylated rabbit anti-mouse $(Fab')_2$ antibody (E413, Dako) and streptavidin–biotin–peroxidase complex (K377, Dako). Diaminobenzidine tetrahydrochloride (Sigma) was applied to give a brown end-product for visualisation and the slides were counterstained with haematoxylin, dehydrated and mounted in synthetic resin.

Correspondence: P.W. Hamilton.
Received 21 September 1993; and in revised form 21 January 1994.

*Image analysis and 3-D reconstruction*

The system used for image storage and reconstruction was a Kontron VIDAS image analyser (Kontron, Germany). This comprises an IBM AT 386 with a frame grabbing board and digitising tablet. The software for image capture, processing, analysis and 3-D reconstruction was Kontron VIDAS v2.1 and Kontron 3-D reconstruction package v2.0. A user application program in Kontron VIDAS macro language was developed to facilitate the procedure. Digital images were stored on a read/write DPL Optistore 650 megabyte optical disk drive.

The serial sections were previewed to identify glands which were complete within the sectioned volume (Figure 1). Each

**Figure 1** Photomicrographs taken from a series of serial sections demonstrating a colorectal gland (arrow) completely sectioned within the volume. The micrographs can be read from left to right and only alternate sections are illustrated. The darkly staining nuclei in the colorectal epithelium are BrdU positively labelled cells.

slide in the series was orientated and the image of the sectioned gland digitally stored as a 512 × 512 pixel colour image. Orientation was carried out by using the closest fit of the gland in question and the surrounding structures. Sequential histological images were stored on optical disk drive for later retrieval.

Each image in the series was then recalled from disk and analysed for subsequent 3-D reconstruction. For each image, the basement membrane was interactively traced using an on-line cursor overlying the image (Figure 2). The tracing was then stored digitally as a binary image. BrdU-negative cells were then highlighted and their positions stored (Figure 3). Finally, BrdU-positive cells were marked and stored as before (Figure 4). As the original histological images were stored digitally, this allowed direct comparison between sequential pairs of sections to ensure that the same cells were not counted twice.

**Figure 2** For each tissue section, the basement membrane of the gland was traced. This could be seen as a green line overlying the image. This line was then converted into a binary image and entered as a level into the 3-D reconstruction.

**Figure 3** Highlighted negative epithelial cells (green). These cell positions were entered as the same level into the reconstruction but into a different colour channel.

**Figure 4** Highlighted BrdU-positive cells (red). These cell positions were again stored for reconstruction.

*Three-dimensional reconstruction*

The binary images were automatically scanned by the 3-D reconstruction software and entered as a level in the reconstruction. After all images were entered, the complete gland could be reconstructed and stored to disk.

Several means of viewing reconstructed images were available, and images could be rotated to any angle for examination. Data for basement membrane, negative and positive cell coordinates were registered in different channels, allowing separate or combined analysis of reconstructed images (Figures 5 and 6). Colours could be linked to the different channels, improving the analysis of positive and negative BrdU fractions within the crypt (Figure 6). In addition, real-time rotations of the gland could be carried out by image animation.

*Quantitative assessment of the 3-D reconstruction*

The binary images which were used for the 3-D reconstruction could also be subjected to quantitative analysis. Each binary image in the series was automatically analysed using the VIDAS system, and the number of positive and negative BrdU cells which had been recorded were counted. This allowed the following parameters to be measured.

1. Total number of BrdU-positive cells per gland.
2. Total BrdU labelling index (positive/negative + positive cells) per gland.

**Figure 5** Reconstruction of the basement membrane only.

**Figure 6** The use of colours was particularly useful in interpretation of reconstructed images. This illustrates a colorectal gland from a patient with a family history of colorectal cancer. The green points represent BrdU-negative epithelial cells; the red points indicate the position of BrdU-positive cells. Notice that the red cells extend beyond the lower third of the crypt. Images such as this could be rotated in 3-D space and viewed at any angle.

3. Three-dimensional (boxes) could be interactively positioned, allowing the measurement of compartmental indices for the reconstructed gland and an assessment of the pattern of proliferation throughout the gland (Figure 7). The glands could be divided into thirds or fifths on the basis of gland cell numbers counted from the base to the mouth on the most central longitudinal section from the gland.

### Results

The method was time-consuming but provided valuable data on the spatial distribution of proliferation within a gland and on the number of proliferating cells in relation to the entire gland unit. The quantitative data obtained for the gland in Figure 6 are listed in Table I. The fact that this is a hyperplastic gland from a high-risk patient is reflected in the high overall proportion of labelled cells in the gland and the proportion of labelled cells occurring in the middle third of the gland.

**Figure 7** This demonstrates the definition of 3-D windows to identify gland compartments in the calculation of compartmental BrdU labelling indices.

**Table I** Labelling calculations for the reconstructed gland shown in Figure 6. This is a hyperplastic gland from a patient with a family history of colorectal cancer and shows an increased overall labelling index and movement of S-phase cells into the upper compartments of the gland

| | |
|---|---|
| Total cells in gland | 5,854 |
| Total BrdU-labelled cells per gland | 618 |
| Total labelling index | 11% |
| Compartmental labelling indices | |
| Bottom third | 19% |
| Middle third | 13% |
| Top third | 1% |

The number of glands necessary to calculate a representative proliferative value for a particular case is difficult to predict as gland-to-gland variation will depend on the tissue and the nature of the disease being studied. An examination of ten colonic glands from normal low-risk control patients gave a mean number of labelled cells per gland of 115 with a confidence interval of 99–131.

Interpretation of reconstructed images was greatly facilitated by colour coding different image components and by rotation of images.

### Discussion

The study of tissues has been for many years based on tissue section analysis which provides a (mostly) two-dimensional view of a three-dimensional structure. This reduction of dimensions (3-D→2-D), results in lost information and leads to observations which are biased (Howard, 1990). This is almost certainly the case in many studies examining cell proliferation in gastrointestinal mucosa from tissue sections.

It is clear, therefore, that a 3-D approach to the analysis of cell proliferation in gastrointestinal epithelium is necessary not only in the quantitative analysis of gland cell proliferation, but also in understanding the spatial relationship between proliferating cells and gland architecture. The gland microdissection technique described now in a number of publications (Clarke, 1970; Ferguson *et al.*, 1977; Goodlad *et al.*, 1991) represents a powerful method for examining the total gland cell population. Relating the number of cells to the gland compartment in this way has been shown to be much more sensitive to proliferative changes than the calculation of a labelling index.

The computerised 3-D reconstruction method described in the current paper represents an alternative method of quantifying proliferative changes and expressing these as total labelled cells per gland. However, in contrast to the microdissection approach, fresh tissue samples are not required and the method can be used to examine stored paraffin-embedded tissue. In addition, the method can be applied to analysis of any marker for glandular proliferating cells that can be identified on tissue sections, e.g. mitotic figures, Ki67, BrdU and proliferating cell nuclear antigen (PCNA) immunohistochemistry. Antibodies such as PC10 and MIB1 are proported to label proliferating cells in formalin-fixed, paraffin-embedded tissue, so allowing the 3-D analysis of cell proliferation in retrospective material. In essence, the spatial distribution of any marked cell within gastrointestinal glands can be examined in three dimensions using the methodology outlined here.

An important addition to the software was the ability to calculate compartmental indices provided both the labelled and non-labelled cells are counted. This permits a more accurate measure of alterations in the spatial distribution of proliferating cells along the length of the gland and this, in combination with a 3-D visual perspective, represents a valuable analytical tool in the study of disease. Visualising the positional distribution within the crypt is enhanced by also being able to view the basement membrane in the reconstructed object.

The number of glands required to obtain a statistically meaningful result depends on the variation in labelled cells between glands. In glands from normal controls this was shown to be quite high, although this depends on how small a change one wishes to detect. Variation is likely to increase in diseased colorectal glands. Goodlad *et al.* (1991) report counting 15–20 glands per case using the microdissection technique. This would be possible using the current method but would be a time-consuming process. After serial sections have been cut, assessing the same number of glands using this computerised technique could take as long as 6 h. The current approach, therefore, does not represent a method which can be rapidly applied in the clinical setting but is more suitable as an analytical and quantitative research tool which has distinct advantages. The effort involved in reconstructing glands can be reduced by counting only the positively labelled cell population and expressing this as number per gland. Counting only the positive cells, however, precludes the calculation of compartmental indices unless the gland is divided on the basis of its length or volume and positive cells expressed as a number per compartment.

Visualisation of 3-D changes in glandular architecture and shape is important, and using serial sections possible changes introduced by removal of the gland from its surrounding tissue are avoided. Previous workers have shown that the examination of glandular structure in three dimensions provides additional useful information in the study of gastrointestinal neoplastic lesions (Takahashi & Iwama, 1984*a*,*b*; Campbell *et al.*, 1992). However, the current approach allows glandular architecture to be examined in association with cell proliferation, and this may provide additional useful information on the proliferative and structural development of gastrointestinal neoplasia from its early stages. An important potential use of the 3-D technique therefore lies in the analysis of tortuous or branching glands which are not easily microdissected (Goodlad *et al.*, 1991). Such glands are found in hyperplastic and adenomatous colorectal mucosa, and reconstruction techniques might provide a valuable insight into the structural and proliferative characteristics of such lesions in so far as the glands can be followed in 3-D space on serial sections. This method should also be of use in the analysis of proliferation in gastric fundal glands whose complexity makes microdissection difficult (Goodlad *et al.*, 1991).

The effort required increases with gland complexity. In adenomatous lesions, not only does the size of the glands increase, requiring the examination and reconstruction of a larger number of tissue sections, but the reconstructed image becomes more difficult to interpret. The value of image rotation by animation and viewing the process of reconstruction (i.e. the piling up of sequential profiles) on the computer screen cannot be overemphasised. This provides a much clearer insight into the spatial relationship of tissue structures compared with static images of the reconstructed object (even if surface rendering and shading algorithms are used) and so enhances the perception of the third dimension. Three-dimensional reconstructions of complex objects are therefore difficult to convey graphically in journal articles such as this.

Of course, the method described in this paper is not restricted to the analysis of cell proliferation markers. It may provide a more accurate means of assessing apoptosis, cell differentiation, gene expression and other genotypic and phenotypic markers in relation to the gland unit. Deciphering the intricate cellular organisation seen within the gastrointestinal gland in three dimensions is fundamental to our understanding of neoplasia and its detection at an early stage. This method provides a tool whereby this organisation can be better understood.

Future work should examine ways to increase the speed of serial section analysis either by automated image analysis or through the use of confocal laser scanning microscopy (CLSM). CLSM allows optical sections of high resolution to be taken through thick blocks of tissue, removing the need for the time-consuming task of physical tissue sectioning and section orientation. The equipment, however, is expensive, and immunohistochemical methods need to be adapted and fully investigated for thick tissue sections. The benefit of computer-based reconstruction of serial physical sections is that the equipment costs a fraction of the price of a CLSM and standard immunohistochemistry can be applied. Nevertheless, the advantages of CLSM in 3-D analysis have been demonstrated in several areas (Agard, 1984; Baak *et al.*, 1987; Brakenhoff *et al.*, 1988; Kett *et al.*, 1992) and are currently being investigated by this group as a means of 3-D reconstruction in colorectal gland analysis.

This work was funded by a DHSS (NI) research grant the Ulster Cancer Foundation, Medical Research Council, Action Cancer, Royal College of Surgeons of Edinburgh, The Queen's University of Belfast and the Royal Victoria Hospital, Belfast.

### References

AGARD, D. (1984). Optical sectioning microscopy: cellular architecture in three dimensions. *Ann. Rev. Biophys. Bioeng.*, **13**, 191–219.

BAAK, J.P.A., THUNNISSEN, F.B.J.M., OUDEJANS, C.B.M. & SCHIPPER, N.W. (1987). Potential clinical uses of laser scanning microscopy. *Appl. Opt.*, **26**, 3413–3416.

BRAENDGAARD, H. & GUNDERSEN, H.J.G. (1986). The impact of recent stereological advances on quantitative studies of the nervous system. *J. Neurosci. Methods*, **18**, 39–78.

BRAKENHOFF, G.J., VAN DER VOORT, H.T.M., VAN SPRONSEN, E.A. & NANNINGA, N. (1988). Three-dimensional imaging of biological structures by high resolution confocal scanning laser microscopy. *Sacnning Microsc.*, **2**, 33–40.

CAMPBELL, F., GARRAHAN, N.J., DEVERELL, M.H., WHIMSTER, W.F. & WILLIAMS, G.T. (1992). Application of a computer aided design system to the 3 dimensional reconstruction of colonic crypts. *J. Pathol.*, **168** (Suppl.), 125.

CLARKE, R.M. (1970). Mucosal architecture and epithelial cell production rate in the small intestine of the albino rat. *J. Anat.*, **107**, 519–529.

FERGUSON, A., SUTHERLAND, A., MACDONALD, T.T. & ALLAN, F. (1977). Technique for microdissection and measurement in biopsies of human small intestine. *J. Clin. Pathol.*, **30**, 1068–1073.

GOODLAD, R.A., MADGEWICK, A.J.A., MOFFATT, M.R., LEVIN, S., ALLEN, J.L. & WRIGHT, N.A. (1989). Prostaglandins and gastric epithelium: effects of misoprostol on gastric epithelial cell proliferation in the dog. *Gut*, **30**, 316–321.

GOODLAD, R.A., LEVI, S., LEE, C.Y., MANDIR, N., HODGSON, H. & WRIGHT, N. (1991). Morphometry and cell proliferation in endoscopic biopsies: evaluation of a technique. *Gastroenterology*, **101**, 1235–1241.

HOWARD, V. (1990). Stereological techniques in biological electron microscopy. In *Biophysical Electron Microscopy*, Hawkes, P.W. & Valdre, U. (eds), pp. 479–508. Academic Press: London.

KETT, P., GEIGER, B., EHEMANN, V. & KOMITOWSKI, D. (1992). Three-dimensional analysis of cell nucleus structures visualised by confocal scanning microscopy. *J. Microsc.*, **167**, 169–179.

SOKAL, R.R. & ROHLF, F.J. (1981). *Biometry. The Principles and Practice of Statistics in Biological Research*. W.H. Freeman: New York.

TAKAHASHI, T. & IWAMA, N. (1984*a*). Architectural pattern of gastric adenocarcinoma. A 3-dimensional reconstruction study. *Virchows Archiv. (Pathol. Anat.)*, **403**, 127–134.

TAKAHASHI, T. & IWAMA, N. (1984*b*). Atypical glands in gastric adenoma. Three dimensional architecture compared with carcinomatous and metaplastic glands. *Virchows Archiv. (Pathol. Anat.)*, **403**, 135–148.

TANNOCK, I.F. (1967). A comparison of the relative efficiencies of various metaphase arrest agents. *Exp. Cell. Res.*, **47**, 345–356.

WRIGHT, N. & ALISON, M. (1984). *The Biology of Epithelial Cell Populations*, Vol. 2. Clarendon Press: Oxford.

WRIGHT, N.A., CARTER, J. & IRWIN, M. (1989). The measurement of villus cell population size in the mouse small intestine in normal and abnormal states: a comparison of absolute measurements with morphometric estimators in sectioned immersion-fixed material. *Cell. Tissue Kinet.*, **22**, 425–450.